# Preparation of *trans*-Crocetin with High Solubility, Stability, and Oral Bioavailability by Incorporation into Three Types of Cyclodextrins

**DOI:** 10.3390/pharmaceutics15122790

**Published:** 2023-12-16

**Authors:** Nan Liu, Jie Xiao, Ling-He Zang, Peng Quan, Dong-Chun Liu

**Affiliations:** 1 School of Pharmacy, Shenyang Pharmaceutical University, Shenyang 110016, China; 101030221@syphu.edu.cn (N.L.); quanpeng@syphu.edu.cn (P.Q.); 2 School of Chinese Materia Medica, Shenyang Pharmaceutical University, Shenyang 110016, China; 19950735397@163.com; 3 School of Life Science and Biopharmaceutics, Shenyang Pharmaceutical University, Shenyang 110016, China; 104040412@syphu.edu.cn

**Keywords:** crocetin, inclusion complex, solubility, dissolution, stability, oral bioavailability

## Abstract

Crocetin (CRT), an active compound isolated from saffron, exhibits several pharmacological activities, including anti-tumor and immune-regulatory activities, and is effective against myocardial ischemia and coronary heart disease; however, its low stability and solubility limit its clinical application. Therefore, we investigated CRT inclusion complexes (ICs) with three cyclodextrins—α-CD, HP-β-CD, and γ-CD—suitable for oral administration prepared using an ultrasonic method. Fourier transform infrared spectroscopy and powder X-ray diffraction indicated that the crystalline state of CRT in ICs disappeared, and intermolecular interactions were observed between CRT and CDs. ^1^H nuclear magnetic resonance and phase solubility studies confirmed CRT encapsulation in the CD cavity and the formation of ICs. In addition, we observed the morphology of ICs using scanning electron microscopy. All ICs showed a high drug encapsulation efficiency (approximately 90%) with 6500–10,000 times better solubilities than those of the pure drug. CRT showed rapid dissolution, whereas pure CRT was water-insoluble. The formation of ICs significantly improved the storage stability of CRT under heat, light, and moisture conditions. Further, the peak time of CRT in rats significantly decreased, and the relative bioavailability increased by approximately 3–4 times. In addition, the oral bioavailability of CRT IC was evaluated. Notably, the absorption rate and degree of the drug in rats were improved. This study illustrated the potential applications of CRT/CD ICs in the food, healthcare, and pharmaceutical industries, owing to their favorable dissolution, solubility, stability, and oral bioavailability.

## 1. Introduction

Saffron, which is widely used in foods, cosmetic ingredients, and phytopharmaceuticals, is extracted from the dried stigmas of *Crocus sativus* L., which belongs to the family Iridaceae [1,2,3,4]. Saffron is used in both folk and modern medicines [5,6,7,8]. Crocin is one of the major bioactive constituents of saffron that is favored for oral administration owing to its water-solubility, safety, and lack of toxicity or side effects [9,10,11]. Crocin is converted into its active metabolite, crocetin (CRT), in the intestine after oral administration to exert pharmacological effects [12]. The direct administration of CRT can potentially yield improved pharmacological effects.

CRT, extracted and separated from the stigmas of *C. sastivus* [13,14], exhibits several pharmacological activities, such as anti-tumor, heart protection, memory enhancement, anti-anxiety, and anti-depression activities, and is effective against Alzheimer’s disease, myocardial ischemia, and coronary heart diseases [15,16,17]. CRT has two isomers—*cis* and *trans*—and its pharmacological efficacy is attributed primarily to the *trans*-isomer [18]. However, CRT is sensitive to heat, light, and pH and is insoluble in water and most organic solvents [19]. It partially dissolves in pyridine, dimethyl sulfoxide, and alkaline aqueous solutions above pH 9.0 [20]. Poor stability and solubility are major obstacles in its formulation and limit the pharmaceutical applications of CRT. Most formulation methods use solvents to encapsulate drugs; however, for CRT, suitable encapsulation formulations and preparation methods are extremely limited. In addition, improving the water solubility and bioavailability of CRT is crucial for maximum pharmacological efficacy. Several reports have evaluated various delivery approaches for CRT, including nanoliposomes, microencapsulation, and lipid nanoparticles; however, few studies have focused on improving the stability, water solubility, and bioavailability of CRT [21,22,23]. Li et al. used β-cyclodextrin-based nanosponges to improve the solubility of CRT, obtaining an elevated aqueous solubility of 7.27 ± 1.11 μg/mL [24]. Wong et al. developed an effective method to prepare CRT–γ-CD inclusion complexes (ICs) with enhanced CRT bioavailability and pharmacological effects against Alzheimer’s disease after intravenous injection; however, the authors did not describe the oral bioavailability of CRT [25]. Research aimed at improving the storage stability and oral bioavailability of CRT remains lacking.

Cyclodextrin (CD) is an effective drug carrier for CRT [24,25] with a wide range of applications in the agrochemical, pharmaceutical, fragrance, and food industries owing to its complexation ability and other versatile characteristics [26,27]. In addition to naturally occurring CDs (α, β, and γ-CD), modified CDs, including HP-β-CD, RM-β-CD, and SBE-β-CD, have attracted substantial attention in the pharmaceutical industry [28,29]. CDs are commonly used to enhance the water solubility, bioavailability, and stability of guest molecules while maintaining their pharmacological properties after forming ICs [30]. Moreover, CDs can mask the pungent odor and bitterness of drugs, making them ideal for the development of pharmaceutics and healthy food products.

Oral administration has several advantages over other routes, including the lack of damage to the skin or mucous membranes, low cost, and convenient storage. In addition, orally administered drugs are better tolerated by patients. Aiming to increase the oral bioavailability of CRT, this study used a *trans*-isomer of CRT along with two natural CDs (α and γ-CD) and a modified CD (HP-β-CD) to prepare three solid CRT/CD ICs using sonication and freeze-drying. The ICs were characterized based on the molecular states of each component. In addition, the water solubility, storage stability, and oral bioavailability of the CRT inclusion complex were investigated.

## 2. Materials and Methods

### 2.1. Materials

**Figure 1:**
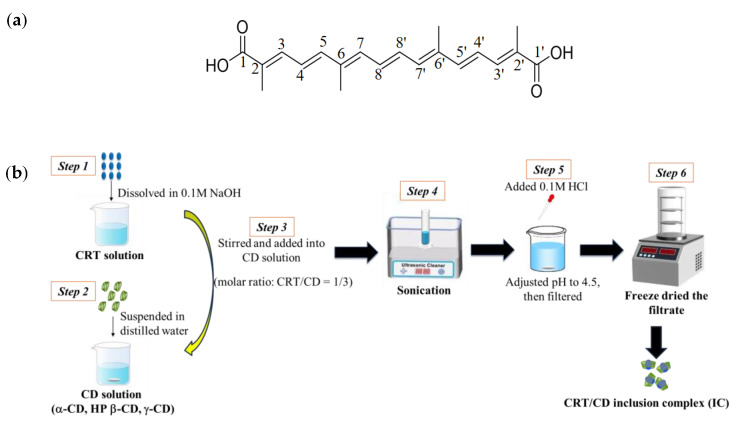
(**a**) Chemical structure of crocetin (CRT). (**b**) Schematic processes for preparing CRT/CD inclusion complex (IC).

The *trans*-isomer of CRT (≥98%) was obtained from saffron and purified in our laboratory using an alkaline hydrolysis via response surface methodology, as previously described [31]. α-CD, HP-β-CD, and γ-CD were purchased from Shanghai Macklin Biochemical Co., Ltd. (Shanghai, China). All chemicals and solvents used in this study were of analytical grade. The chemical structure of CRT is presented in Figure 1a.

### 2.2. Animals

Male Sprague–Dawley (SD) rats (weighing 180–220 g) were purchased from the Laboratory Animal Center of Shenyang Pharmaceutical University (Shenyang, China).

### 2.3. Preparation of CRT/CD Inclusion Complexes

An outline of sample preparation processes is illustrated in Figure 1b. CRT was dissolved into 0.1 M NaOH to make the CRT solution. Each CD sample was suspended in distilled water. Next, the CRT solution was added dropwise to each aqueous CD solution with a molar ratio of CRT to CD of 1:3 for all CDs (α-, HP-β-, and γ-CD). All mixed solutions were sonicated for 3 h and supplemented with 0.1 M HCl to adjust the pH to 4.5. The mixed solutions were filtered through a 0.22 μM microporous filter membrane. The filtrate was freeze-dried to obtain solid CRT/CD ICs. CRT concentrations in ICs were assayed using high-performance liquid chromatography (HPLC) at a detection wavelength of 424 nm. The encapsulation efficiency (EE) of CRT was calculated using the following equation:

Encapsulation efficiency (%) = (Encapsulated CRT in IC/Total CRT added) × 100.

The EEs for CRT/α-CD IC, CRT/HP-β-CD, and CRT/γ-CD IC were 89.20 ± 0.43%, 89.93 ± 0.57%, and 91.90 ± 0.39%, respectively (Table S1).

CRT was mixed with each CD (α-CD, HP-β-CD, and γ-CD) for 3 min using a vortex mixer with molar ratios of 1:3 to obtain the CRT/CD physical mixtures (PMs).

### 2.4. Characterization of CRT/CD ICs

#### 2.4.1. Fourier Transform Infrared (FTIR) Spectroscopy

FTIR spectroscopy was used to evaluate all formulation powders (CRT, α-CD, CRT/α-CD PM, CRT/α-CD IC, HP-β-CD, CRT/HP-β-CD PM, CRT/HP-β-CD IC, γ-CD, CRT/γ-CD PM, and CRT/γ-CD IC). Spectra were recorded in the range of 4000–500 cm^−1^ using an FTIR-650 spectrometer (TIANJIN GANGDONG, Tianjin, China) with 32 scans at a resolution of 4 cm^−1^. Samples were prepared using KBr disks containing 1 mg of the complex in 200 mg of KBr. FTIR spectra were analyzed using OPUS 6.0.

#### 2.4.2. Powder X-ray Diffraction (PXRD)

PXRD was performed using a Bruker D8 Advance powder diffraction system (Bremen, Germany). The X-ray source was CuKa radiation under 40 kV and 30 mA. The scanning range (2θ) was 3–30, and the scan speed was 10/min.

#### 2.4.3. Scanning Electron Microscopy (SEM)

Surface morphologies of all formulation powders were obtained using a Regulus8100 scanning electron microscope (Hitachi, Tokyo, Japan) with an accelerating voltage of 5 kV. All samples were electrically conductive because of the addition of a thin coat of gold for 200 s before being examined.

#### 2.4.4. Solution-State ^1^H Nuclear Magnetic Resonance (NMR) Spectroscopy

^1^H NMR experiments were performed using a Bruker 600 NMR spectrometer (Zurich, Switzerland) with tetramethylsilane as the internal standard and DMSO-d6 and D_2_O as solvents. The chemical shifts are reported in δ (ppm) and standard 5 mm NMR tubes were used. Measurement conditions were as follows: temperature, 25 °C; relaxation delay, 1 s; number of scans, 4.

### 2.5. Phase Solubility Study

The phase solubility of CRT was evaluated according to the method reported by Higuchi and Connors [32]. An excess of the drug (5 mg) was added to 10 mL of phosphate buffer (pH 6.8) with various concentrations of α-CD, HP-β-CD, and γ-CD (0–500 mM) in 25 mL stoppered conical flasks, which were shaken at 37 ± 0.5 °C for 72 h to reach equilibrium. The excess drug was removed via filtration using a 0.22 μM microporous filter membrane, and the drug concentrations were analyzed using HPLC. The assay was performed in triplicate for each CRT/CD system. The amount of CRT dissolved was plotted against the molar concentration of CDs, and assuming 1:1 complex formation, the apparent stability constant *K*c was calculated from phase solubility diagrams using the following equation:

*K*_c_ = slope/S_0_(1 − slope) (1)

where S_0_ is the solubility of CRT in the absence of CDs.

### 2.6. HPLC Analysis (Excluding Pharmacokinetics Study)

Each sample was analyzed using a Waters HPLC system consisting of a 2695–2487 UV detector (Milford, MA, USA). The analysis was carried out on a COSMOSIL-C18 (150 mm × 4.6 mm, 5 μm) column. The mobile phase was a 15:85 (*v*/*v*%) mixture of 3.33% glacial acetic acid aqueous solution and methanol. The flow rate was 1.0 mL/min, the detection wavelength was 424 nm, and the injection volume was 10 μL.

### 2.7. Dissolution Test

The dissolution of CRT, PMs, and ICs was evaluated using the USP (version 26) paddle method with a dissolution tester (Tianjin University Electronics Co., Ltd., Tianjin, China). The dissolution medium was phosphate buffer (pH 6.8, 900 mL); the paddles were rotated at 100 rpm at 37.0 ± 0.5 °C. A sample volume of 5 mL was withdrawn from the dissolution medium at predetermined sampling points, filtered using a 0.22 μM microporous filter membrane, and analyzed via HPLC. All dissolution tests were performed in triplicate.

### 2.8. Solubility Determination

Drug solubility at 25 ± 0.5 °C was determined in water and phosphate buffer (pH 6.8). Excesses of CRT and each IC were added to each fluid and placed in a thermostatic water bath in the dark at 25 ± 0.5 °C with a constant shaking rate of 100 rpm for 72 h. After the equilibrium state was reached, the solutions were centrifuged for 15 min, and 2 mL of the supernatant was diluted with an appropriate amount of methanol. Drug concentrations were determined using HPLC.

### 2.9. Effect of Storage on Stability

All samples were sealed in transparent glass bottles and divided into three groups, each containing CRT/α-CD IC (500 mg), CRT/HP-β-CD IC (500 mg), CRT/γ-CD IC (500 mg), and intact CRT (500 mg). The first group was stored at 60 ± 0.5 °C for 10 days. The second group was stored at 25 ± 0.5 °C with a light intensity of 4500 ± 500 lx for 10 days. The third group was stored at 25 ± 0.5 °C with 75% relative humidity for 10 days. The CRT contents were sampled and measured on days 0, 5, and 10. Each test was repeated at least three times.

### 2.10. Pharmacokinetics Study

Twenty-four SD rats were randomly divided into four groups and administered CRT/α-CD IC, CRT/HP-β-CD IC, CRT/γ-CD IC, or intact CRT. All rats were fasted for 12 h and only provided water before experiments. All samples at a dose of 20 mg/kg body weight were administered intragastrically. Blood samples (0.4 mL) were collected from the orbital venous plexus at predetermined time points (0.083, 0.167, 0.25, 0.5, 1, 2, 4, 6, 8, 12, and 24 h) into tubes containing heparin sodium. The blood samples were centrifuged immediately at 4000 rpm for 15 min; the supernatant layer of plasma was separated and stored at 4 °C for 24 h. CRT in plasma was extracted with 600 μL methanol with an internal standard of 10 μL ATRA from 200 μL plasma samples and centrifuged at 10,000 rpm for 15 min at 4 °C. The supernatant layer was transferred to a new tube, concentrated to remove solvents under the LSE-1K vacuum centrifuge concentrator (JTLIANGYOU, Changzhou, China), and redissolved in 200 μL of methanol for analysis. The concentration of CRT was determined using an HPLC (Waters HPLC system consisting of a 2695–2487 UV detector, Milford, MA, USA) using a COSMOSIL-C18 (150 mm × 4.6 mm, 5 μm) column with a detection wavelength of 424 nm. The injection volume was 20 μL, the column temperature was 35 °C, the mobile phase was methanol with water and glacial acetic acid in a ratio (*v*:*v*:*v*) of 92:7.7:0.3, and the flow rate was 1 mL/min. The pharmacokinetic parameters were calculated using DAS 2.0. Relative bioavailability percentage was calculated as follows [33]:

Relative bioavailability (%) = (AUC_0–∞_ IC/AUC_0–∞_ CRT) × 100 (2)

### 2.11. Statistical Analysis

All results are presented as means ± standard deviation. Student’s *t*-test or one-way analysis of variance was used to evaluate significance.

## 3. Results and Discussion

### 3.1. Characterization of CRT/CD IC

#### 3.1.1. FTIR Spectroscopy

**Figure 2:**
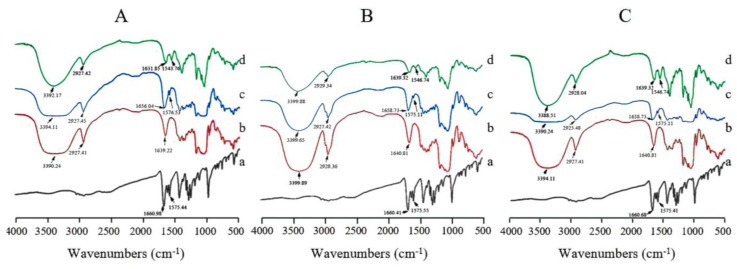
FTIR spectra of (a) CRT, (b) CD, (c) PM, and (d) IC in CRT/α-CD (**A**), CRT/HP-β-CD (**B**), and CRT/γ-CD (**C**) systems.

Various methods were applied to evaluate the formation of ICs. The FTIR spectra of the CRT/α-CD, CRT/HP-β-CD, and CRT/γ-CD systems are represented in Figure 2A–C, respectively. The a, b, c, and d in each spectrum represent pure CRT, natural CD, CRT/CD PM, and CRT/CD IC, respectively. Pure CRT is characterized by the C=O and C=C stretching vibrations at 1660.98 and 1575.44 cm^−1^, respectively [25]. Additionally, several characteristic peaks were observed for the three types of CDs (α-CD, HP-β-CD, and γ-CD), including broad absorption bands due to O-H bonds at 3500–3300 cm^−1^, the C-H stretching vibration at approximately 2930 cm^−1^ on the aromatic ring, and the H-O-H stretching vibration at approximately 1640 cm^−1^. In the CRT/α-CD system, FTIR spectra of CRT, α-CD, CRT/α-CD PM, and CRT/α-CD IC were compared (Figure 2A). The spectrum of PM was a simple superimposition of individual patterns of CRT and α-CD, indicating no interaction between the drug and α-CD [34]. Compared with those for PM, the wavenumbers of C=O and C=C stretching of CRT in IC were shifted to 1631 and 1543 cm^−1^, respectively. Such behaviors could be attributed to the interaction between CRT and α-CD in IC. Moreover, the peak patterns of IC were broader than those of pure CRT and PM due to the interaction between the drug and CD (Figure S1A). Similar changes were observed in the CRT/HP-β-CD and CRT/γ-CD systems, indicating interactions between CRT and both HP-β-CD and γ-CD in the ICs (Figure 2B,C). The characteristic peaks of CRT significantly shifted to lower frequencies in the three ICs, and the peak patterns became broad, possibly due to the interactions between CRT and CDs, such as hydrogen bonding and van der Waals forces [35,36,37]. These findings confirmed the successful formation of CRT/CD ICs.

#### 3.1.2. PXRD Patterns

**Figure 3:**
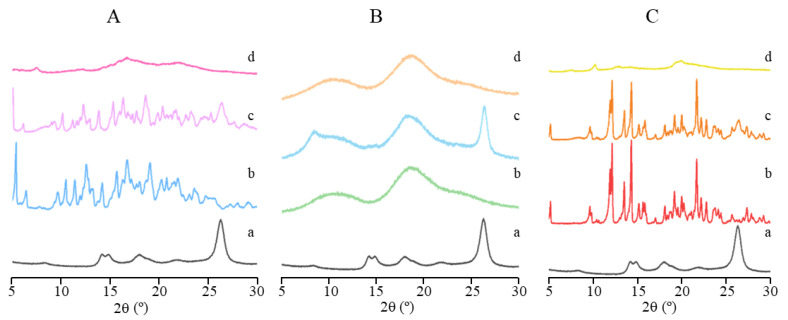
PXRD patterns of (a) CRT, (b) CD, (c) PM, and (d) IC in CRT/α-CD (**A**), CRT/HP-β-CD (**B**), and CRT/γ-CD (**C**) systems.

To determine the solid state of ICs, a PXRD analysis was also performed. PXRD patterns of CRT/α-CD, CRT/HP-β-CD, and CRT/γ-CD systems are represented in Figure 3A–C, respectively. The a, b, c, and d in each pattern represent pure CRT, natural CD, CRT/CD PM, and CRT/CD IC, respectively. Characteristic peaks of crystalline CRT were observed at 14.17, 14.84, 17.83, and 26.31° (2θ), as shown in Figure 3. α-CD and γ-CD exhibited a series of strong and sharp diffraction peaks, indicating that these two natural CDs existed in crystalline form (Figure 3A,C). In contrast, HP-β-CD was amorphous, displaying a diffuse halo pattern, as shown in Figure 3B [38]. In the diffraction patterns of three CRT/CD PMs, peaks derived from the CRT crystal and three natural CDs were observed, which could be a simple superposition of the drug and each CD. Meanwhile, in the case of CRT/CD ICs, CRT/α-CD and CRT/γ-CD ICs mainly exhibited halo patterns, CRT/HP-β-CD ICs exhibited the same amorphous state as that of natural HP-β-CD, and no ICs showed CRT crystalline peaks. These results indicated that the crystalline structure of CRT transformed into an amorphous state in all three ICs, possibly due to the interaction between CRT and CDs [39,40]. This amorphous transformation of CRT in all three ICs was also confirmed using differential scanning calorimetry (Figure S2).

#### 3.1.3. SEM

**Figure 4:**
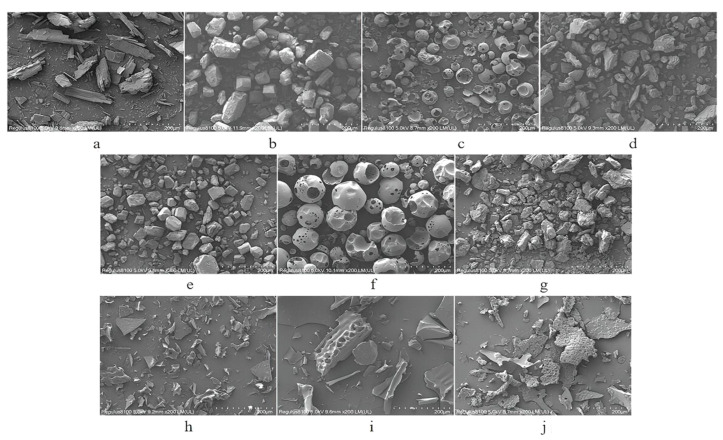
SEM of (**a**) CRT, (**b**) α-CD, (**c**) HP-β-CD, (**d**) γ-CD, (**e**) CRT/α-CD PM, (**f**) CRT/HP-β-CD PM, (**g**) CRT/γ-CD PM, (**h**) CRT/α-CD IC, (**i**) CRT/HP-β-CD IC, and (**j**) CRT/γ-CD IC.

The surface textures of the CRT, CDs, PMs, and ICs are shown in Figure 4. SEM images in Figure 4a–d represent the crystalline states of pure CRT, natural α-CD, natural HP-β-CD, and natural γ-CD, respectively. Figure 4e–g represent the PMs of CRT/α-CD, CRT/HP-β-CD, and CRT/γ-CD, respectively. Figure 4h–j represent the ICs of CRT/α-CD, CRT/HP-β-CD, and CRT/γ-CD, respectively. Crystalline CRT existed in irregularly prismatic structures, natural α-CD appeared as regular cubic structures, natural HP-β-CD exhibited spherical particles with cavity structures, and natural γ-CD showed a block-like shape with a smooth surface (Figure 4a–d). The micrograph of all three CRT/CD PMs showed a mix of the original morphology of CRT and natural CDs, with no new structures (Figure 4e–g). Conversely, the CRT/CD ICs differed from CRT, α-CD, HP-β-CD, and γ-CD in terms of particle appearance, shape, and size, and the original morphology of CRT and the raw CD materials disappeared (Figure 4h–j). Such modifications in particle shape and aspect are consistent with the interactions between CRT and CD in ICs [41,42].

#### 3.1.4. ^1^H NMR Spectra

**Figure 5:**
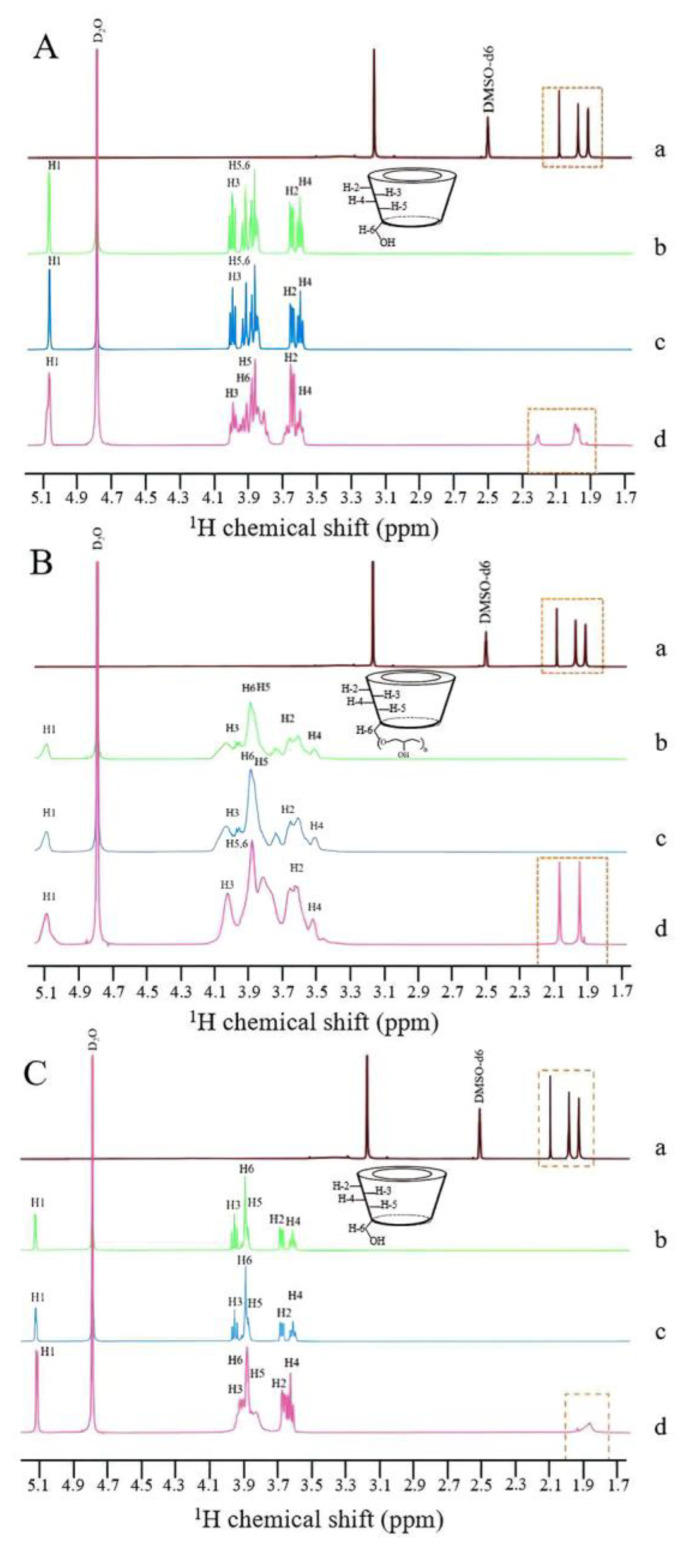
^1^H NMR spectra of (a) CRT (DMSO-d_6_), (b) CD (D_2_O), (c) PM (D_2_O), and (d) IC (D_2_O) in CRT/α-CD (**A**), CRT/HP-β-CD (**B**), and CRT/γ-CD (**C**) systems from 1.7 to 5.1 ppm.

ICs were also characterized using solution-state ^1^H NMR spectroscopy. Guest drug encapsulation into the cavity of CDs can be observed by the chemical shifts of protons in the ^1^H-NMR spectrum [43]. Figure 1a and Figure 5 show the structure of the CRT molecule and ^1^H NMR spectra of CRT, CDs, PMs, and ICs. The chemical shifts of CRT in CRT/CD PMs and ICs were investigated. CRT was detected in DMSO-d_6_ and D_2_O solvents using ^1^H NMR spectroscopy (Figure S3). The natural CDs, CRT/CD PMs, and ICs were detected only in the D_2_O solution using ^1^H NMR spectroscopy (Figure 5A(b–d), Figure 5B(b–d), and Figure 5C(b–d), respectively). As CRT dissolved in DMSO-d_6_, characteristic peaks were detected in the ^1^H-NMR spectrum at 1.9–2.1 ppm based on the characteristic peaks of sharp aliphatic H_2,2′,6,6′_ [44]. However, due to the poor solubility of CRT in the D_2_O solution, ^1^H peaks were not observed. Only the D_2_O peak at 4.79 ppm was observed (Figure S3) [45]. No characteristic peaks of CRT were detected in ^1^H spectra of the three CRT/CD PMs, indicating that the solubility of CRT in D_2_O was extremely low for PMs. The simple PM of CRT and CD showed poor CRT solubilization, with no interaction between CRT and CD in PM. In contrast, for the three CRT/CD ICs, peaks were detected in the range of 1.7–2.3 ppm, which were not detected for natural CD or PMs. According to the ^1^H spectrum of CRT in DMSO-d_6_ (Figure 5A(a)) and previous findings [44], these peaks (1.7–2.3 ppm) may correspond to the sharp aliphatic H_2,2′,6,6′_ of CRT, indicating that the solubility of CRT in D_2_O was improved due to the formation of IC. In addition, variation in CDs in CRT/CD PMs and ICs was also evaluated, and ^1^H chemical shifts are shown in Table 1. Δδ (Δδ_CD_−_PM_ = δ_CD_−δ_PM_ and Δ_δCD_−_IC_ = δ_CD_−δ_IC_) was defined as the change in chemical shift between the raw CD and CRT/CD PM or CRT/CD IC. H_1_–H_6_ protons located on the surface of the three CDs showed slight or no chemical shifts in their PMs, indicating no interactions between CRT and CDs and that CRT did not enter the CD cavities in PMs [46]. In contrast, for the three CRT/CD ICs, slight changes were observed in the chemical shifts of H_1_, H_2_, and H_4_ protons located on the outer surface of CDs, along with significant changes in the chemical shifts of H_3_ and H_5_ protons located on the inner surface of CDs. The up-field shifts of H_3_ and H_5_ protons might be explained by van der Waals interactions between CRT and the inner surface of CDs [47]. In the stereoscopic structure of CD, the interior H_3_ proton is located on the wide side of the CD cavity and the H_5_ proton is located on the narrow side of the cavity. The chemical shifts of H_3_ in the α-CD and γ-CD cavities were larger than those of H_5_. These results suggested that CRT entered the cavities of both α-CD and γ-CD from the wide side. However, the chemical shifts of H_3_ in the HP-β-CD cavity were smaller than those of H_5_, possibly attributed to the entry of CRT from the narrow side [48,49]. Among these three CDs, HP-β-CD showed relatively larger changes for H_3_ and H_5_ protons than the other two natural CDs (α- and γ-CD), suggesting a stronger interaction with CRT. The significant changes in the chemical shift of CD protons as well as the detection of aliphatic protons of CRT only in CRT/CD ICs demonstrated that CRT was successfully encapsulated into the cavity of CDs and formed ICs [50,51,52]. Combined with the above FTIR, PXRD, and NMR results, these results confirmed that CRT formed ICs with the three types of CDs.

### 3.2. Phase Solubility Study

**Figure 6:**
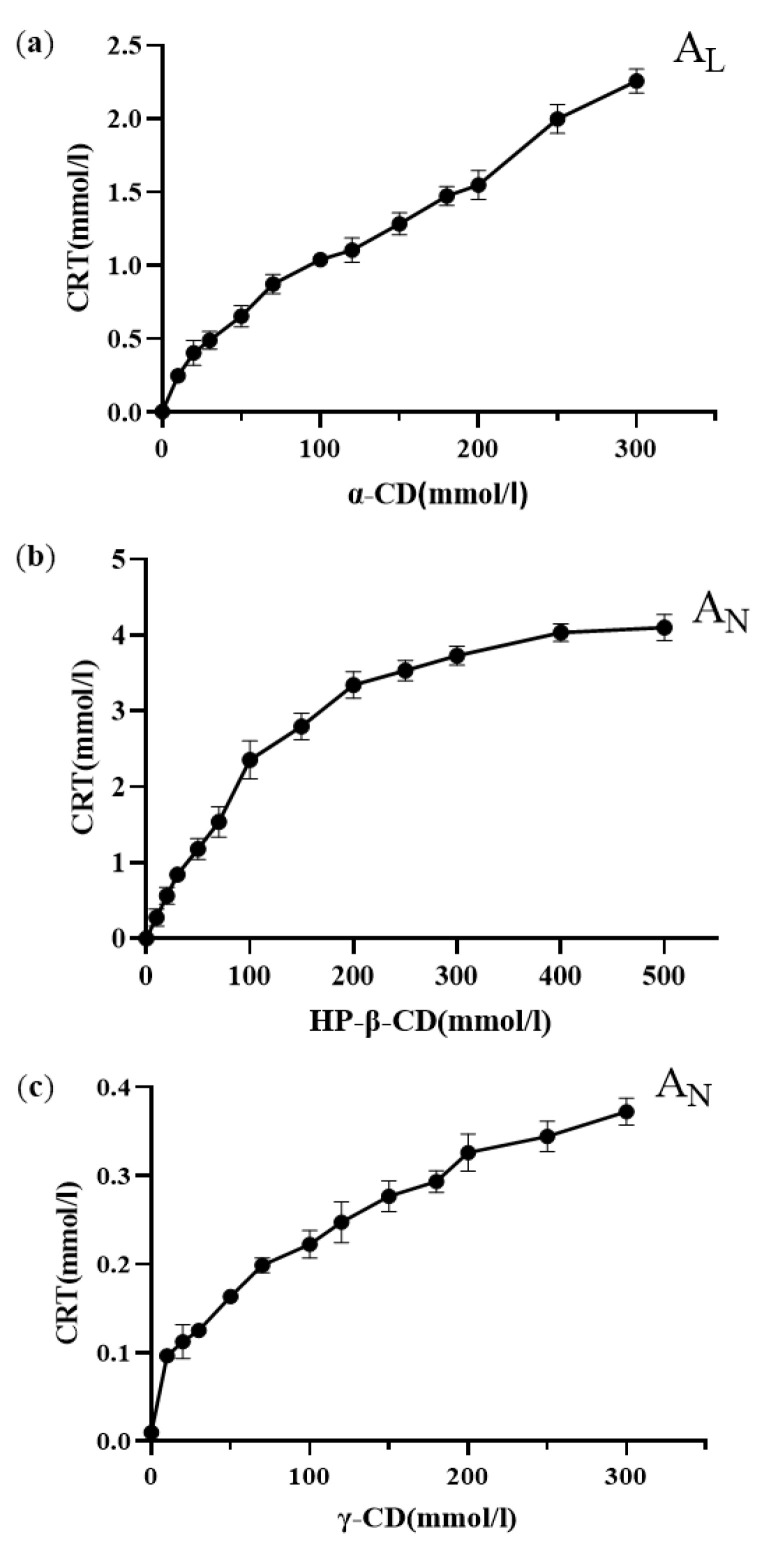
Phase solubility diagrams of (**a**) CRT/α-CD IC, (**b**) CRT/HP-β-CD IC, and (**c**) CRT/γ-CD IC (n = 3, mean ± S.D.).

The phase solubility curves of CRT in α-CD, HP-β-CD, and γ-CD at 37 ± 0.5 °C in phosphate buffer (pH 6.8) are shown in Figure 6a–c. The solubility of CRT increased linearly as the CD concentration increased for α-CD, whereas the solubility of CRT in HP-β-CD and γ-CD increased nonlinearly and deviated from the straight line in the negative direction as the CD concentrations increased. This indicated that soluble ICs formed for all three types of CDs. According to the Higuchi and Connors classification [32], these diagrams could be classified as A_L_-type (linear increase in drug solubility as a function of cyclodextrin concentration) for α-CD and A_N_-type (negatively deviating isotherm) for HP-β-CD and γ-CD [53]. It was speculated that the inclusion ratios of CRT to CD may be 1:1 for α-CD and 1:2 for both HP-β-CD and γ-CD. Combined with the results of ^1^H NMR, changes in the chemical shifts of H_3_ and H_5_ for CRT/α-CD IC were relatively small; thus, CRT was only partially incorporated in α-CD, and the drug did not fully penetrate into its cavity. As listed in Table S2, the ΔG values of the three ICs were all negative, indicating that the inclusion reactions occur spontaneously. Based on the initial linear part of the profile, the stability constants (*K*_c_) were 3027, 7912, and 427 M^−1^ for α-CD, HP-β-CD, and γ-CD, respectively (Table S2). The stability constant of the IC formed between CRT and HP-β-CD was higher than that of the IC formed with the other two CDs, indicating that the IC formed between CRT and HP-β-CD was more stable, which could be due to the greater water solubility and higher wetting and complexing ability of HP-β-CD. The *K*_c_ of the CRT/γ-CD IC was lower than those of the other two ICs, possibly because γ-CD exhibits a larger pore size and CRT was more likely to dissociate from the CD cavity, resulting in the lowest stability among the ICs [49]. As determined using the continuous variation method [54] (Figure S4), the inclusion ratios were 1:1 for CRT/α-CD IC and 1:2 for both CRT/HP-β-CD and CRT/γ-CD ICs, consistent with the phase solubility results.

### 3.3. Dissolution and Solubility of CRT/CD ICs

**Figure 7:**
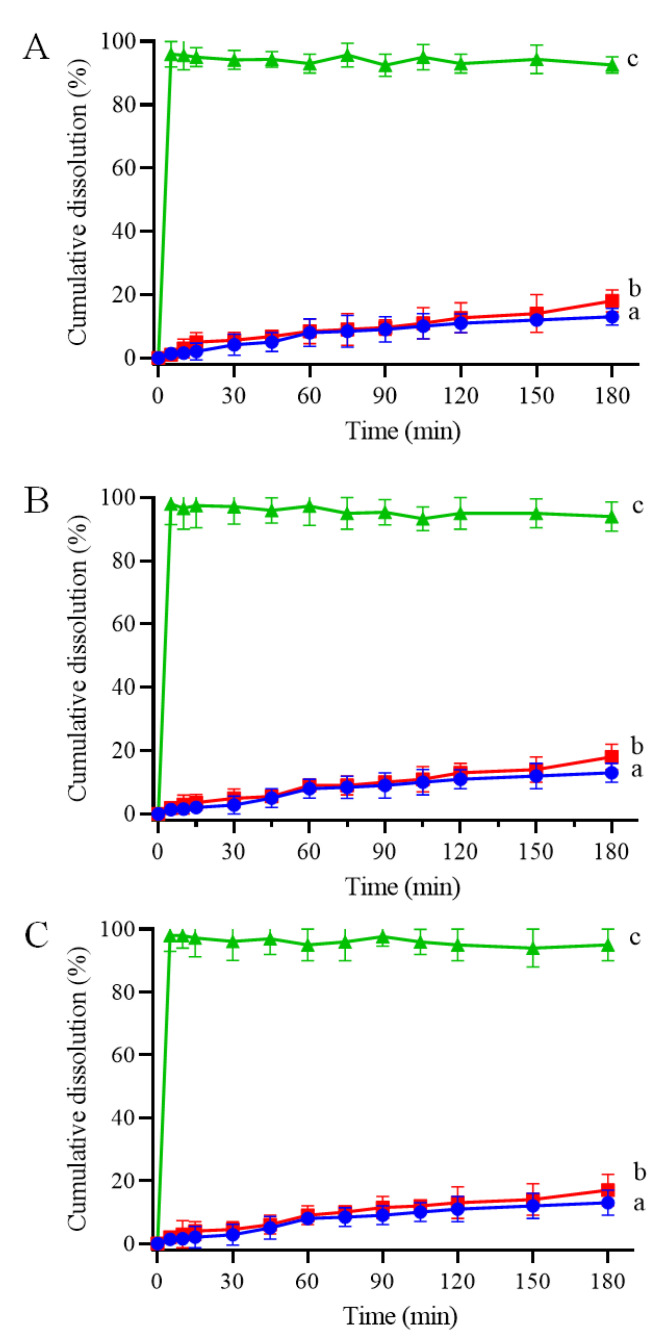
Dissolution profiles of (a) CRT, (b) PM, and (c) IC in CRT/α-CD (**A**), CRT/HP-β-CD system (**B**), and CRT/γ-CD (**C**) systems in phosphate-buffered solution at 37 °C and pH 6.8 (n = 3, mean ± S.D.).

CRT is insoluble in water and most organic solvents; therefore, to determine whether the IC formation improves its dissolution behavior, dissolution properties in phosphate buffer (pH 6.8) were investigated. The CRT dissolution profiles of crystalline CRT, PMs, and ICs of CRT/α-CD, CRT/HP-β-CD, and CRT/γ-CD systems are shown in Figure 7A–C, respectively. The cumulative dissolution rate of crystalline CRT was only 13% at 180 min. Additionally, the dissolution rate of the PMs did not differ substantially from that of crystalline CRT, and the cumulative dissolution rates were 16, 18, and 19% at 180 min for CRT/α-CD, CRT/HP-β-CD, and CRT/γ-CD, respectively. The dissolution rates for both crystalline CRT and PMs were extremely low. Conversely, the cumulative dissolution of CRT from CRT/CD ICs at a sampling time of 5 min was approximately 97%, demonstrating much higher dissolution rates than those of PMs and crystalline CRT. The better solubility of CRT observed in ^1^H NMR and phase solubility studies might explain the fast dissolution of CRT/CD ICs (Figure 5 and Figure 6). In addition, the improvement in dissolution could be largely attributed to the amorphization of CRT after complexation, as indicated by the PXRD results (Figure 3). Solubility tests were performed to compare CRT/CD ICs and pure CRT. The solubility values of the prepared ICs with different CDs are listed in Table 2. The solubilities of pure CRT in water (1.23 ± 0.07 mg/L) and buffer salts (1.84 ± 0.11 mg/L) were extremely low. The solubility of CRT after forming ICs increased by approximately 6500–10,000 times (Table 2). Notably, the best solubility was observed for CRT/HP-β-CD IC, possibly because HP-β-CD, a modified CD, has the best water solubility among the three CDs. Overall, all three CRT/CD ICs conferred an enhanced CRT dissolution rate and solubility, revealing their potential use in the development of effective strategies to enhance the dissolution rate of poorly water-soluble CRT.

### 3.4. Effect of Storage on the Stability of CRT

**Figure 8:**
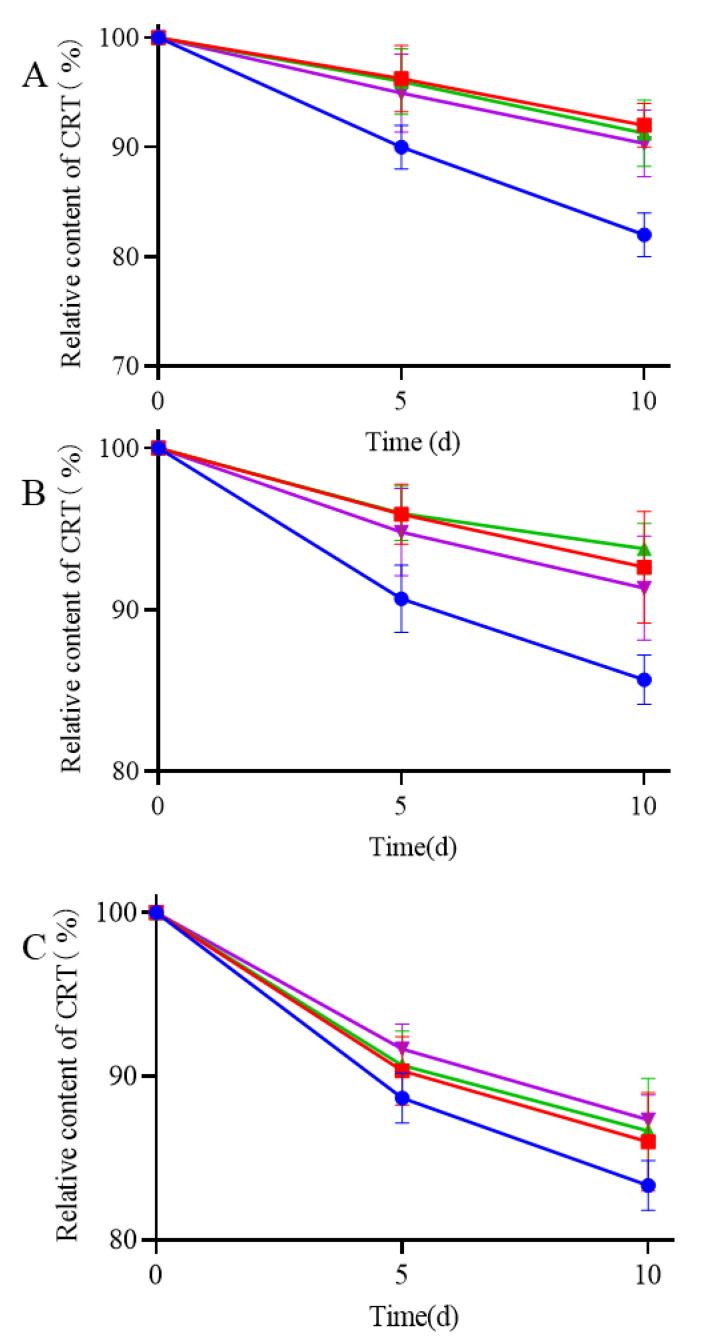
Stability experiments of (●) CRT, (■) CRT/α-CD IC, (▲) CRT/HP-β-CD IC, and (▼) CRT/γ-CD IC at 60 ± 0.5 °C (**A**), light intensity of 4500 ± 500 lx under 25 ± 0.5 °C (**B**), and 75% relative humidity conditions under 25 ± 0.5 °C (**C**).

Stability was evaluated under various heat, light, and moisture conditions to investigate the effect of storage on the CRT content. The degradation curves of all samples (pure CRT, CRT/α-CD IC, CRT/HP-β-CD IC, and CRT/γ-CD IC) were determined by plotting the relative CRT content versus storage time (days) under different conditions. The storage conditions under heat, light, and moisture treatments are shown in Figure 8A–C. The relative contents of CRT and three types of ICs decreased to varying extents under all storage conditions. During thermal treatment (60 ± 0.5 °C) of CRT, the relative content of pure CRT significantly decreased to 80%, whereas ICs exhibited at least 90% retention even after 10 days. The preparation of ICs can significantly mitigate the decrease in CRT and improve thermal instability. During storage at a light intensity of 4500 ± 500 lx at 25 °C, the retention of CRT in ICs was at least 85%, slightly higher than that of pure CRT, indicating that ICs had greater stability than that of pure CRT under light conditions. During storage at an RH of 75% at 25 °C, the retention of both pure CRT and ICs decreased, although the decrease was slightly lower for ICs than pure CRT. CD may be a hydrophilic excipient that exhibits hygroscopicity under highly humid conditions. The encapsulation of water molecules affected the interaction between CD and CRT, resulting in a decrease in CRT contents in ICs [55]. During storage, CRT was degraded under heat, light, and moisture, whereas the formation of ICs could protect CRT from damage caused by these factors.

### 3.5. Pharmacokinetics Study of CRT/CD ICs

**Figure 9:**
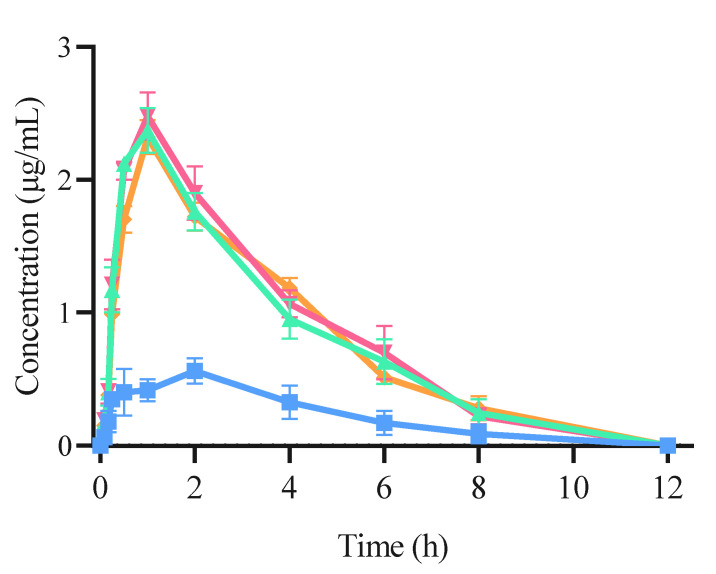
Concentration–time curve of CRT pharmacokinetic profile using SD rats from (■) CRT, (▲) CRT/α-CD IC, (▼) CRT/HP-β-CD IC, and (◆) CRT/γ-CD IC (n = 6, mean ± S.D.).

The plasma concentration–time curves following the intragastric administration of both free CRT and CRT/CD ICs are shown in Figure 9, and the main pharmacokinetic parameters are listed in Table 3. The pharmacokinetic curves of the three CRT/CD ICs were significantly better than those of free CRT, indicating that IC formation increased blood CRT concentration in rats. The peak concentration (C_max_) was 0.545 ± 0.023 μg/mL for free CRT, compared with 2.376 ± 0.118, 2.487 ± 0.126, and 2.355 ± 0.095 μg/mL for CRT/α-CD IC, CRT/HP-β-CD IC, and CRT/γ-CD IC, respectively. In addition, following oral administration (20 mg/kg), the time to reach the maximum concentration (T_max_) was 2 h for free CRT and 1 h for all three CRT/CD ICs.

Therefore, compared with free CRT, the three ICs significantly decreased the peak time of CRT in rats, and the relative bioavailability of the three ICs using α-CD, HP-β-CD, and γ-CD was increased by 4.35, 4.49, and 4.37 times, respectively. Therefore, CRT/CD IC significantly increased the peak concentration, absorption rate, and degree of CRT in vivo, consistent with the in vivo results for various IC formulations [33,56,57]. As calculated using Equation (2), the mean AUC_0-∞_ values of free CRT, CRT/α-CD IC, CRT/HP-β-CD IC, and CRT/γ-CD IC were 2.665 ± 0.196, 9.723 ± 0.222, 10.237 ± 0.343, and 9.869 ± 0.245 μg·h/mL (*p* < 0.01), respectively. This indicated that the CRT/CD ICs could significantly enhance the degree and rate of intestinal absorption of CRT. The bioavailability of free CRT was relatively low, possibly due to its poor solubility in water. The dissolution and absorption in the intestine were both low, lowering the drug contents in vivo. In contrast, the IC formation could improve the solubility of CRT, increase the amount of CRT dissolved in the intestine, and increase the amount of CRT entering the blood from the intestine. These results demonstrated that the oral relative bioavailability of CRT was increased by 3.68, 3.94, and 3.77 times when administrated as IC using α-CD, HP-β-CD, and γ-CD, respectively. CRT/HP-β-CD IC showed a slightly higher relative bioavailability than those of CRT/α-CD and CRT/γ-CD ICs, roughly analogous to the results of the solubility test. In a previous study, the highest plasma concentration of CRT was 1249.3 ± 788.1 ng/mL at approximately 2 h after oral administration in mice, even for a dose of 117.2 mg/mL [58]. Moreover, CRT or saffron tea administered orally to humans showed a T_max_ concentration at approximately 4.6 h, and the concentrations of CRT in the blood ranged from 100.9 to 279.7 ng/mL based on the dose [59,60]. CRT/CD ICs could significantly reduce CRT dose to achieve a higher plasma concentration and shorten T_max_ than those obtained in previous studies after oral administration.

## 4. Conclusions

In this study, we successfully prepared CRT/CD ICs using two natural CDs (α-, and γ-CD) and a modified CD (HP-β-CD) with lower toxicity than β-CD using the sonication method. Notably, we observed enhanced solubility, stability, and bioavailability of CRT. The formation of all three ICs was confirmed using FTIR, PXRD, SEM, and ^1^H NMR. Solubility and dissolution tests indicated that the three CRT/CD ICs significantly improved the water solubility of CRT. Further, IC formation can improve the stability of CRT during storage under heat, light, and moisture conditions. The inclusion ratios determined using phase solubility diagrams and the continuous variation method were 1:1 for CRT/α-CD IC and 1:2 for both CRT/HP-β-CD and CRT/γ-CD ICs. These three ICs of the CRT showed significantly lower peak times than those of pure CRT in rats, and the relative bioavailability of the three ICs using α-CD, HP-β-CD, and γ-CD was increased by 3.68, 3.94, and 3.77 times, respectively. These findings indicated that ICs can improve the relative bioavailability of CRT substantially in rats. NMR analysis, phase solubility study, and solubility tests revealed a superior inclusion efficiency of HP-β-CD with a higher *K*_c_ value, solubility, and relative bioavailability than the other two. However, α-CD and γ-CD showed certain advantages in terms of security and environmental friendliness. These three CDs were effective in carrying CRT, although further evaluation is warranted in terms of pharmacodynamics and toxicology. The preparation of CRT/CD ICs improved the oral relative bioavailability of CRT, offering a new approach for the development of cost-effective solid formulations or healthy foods based on CRT.

## Figures and Tables

**Table 1:** ^1^H chemical shifts of CD in CRT/α-CD (A), CRT/HP-β-CD (B), and CRT/γ-CD (C) systems.

	**H**	**Chemical Shift (ppm)**				
		δ (α-CD)	δ (PM)	Δ δ(CD−PM)	δ (IC)	Δ δ(CD−IC)
	H1	5.067	5.065	0.002	5.070	−0.003
	H2	3.659	3.659	0	3.657	0.002
A	H3	3.998	3.997	0.001	3.993	0.005
	H4	3.601	3.599	0.002	3.598	0.003
	H5	3.886	3.886	0	3.883	0.003
	H6	3.919	3.918	0.001	3.916	0.003
	H	Chemical shift (ppm)				
		δ (HP-β-CD)	δ (PM)	Δ δ(CD−PM)	δ (IC)	Δ δ(CD−IC)
	H1	5.091	5.090	0.001	5.087	0.004
	H2	3.653	3.652	0.001	3.651	0.002
B	H3	4.032	4.033	-0.001	4.022	0.010
	H4	3.518	3.516	0.002	3.521	−0.003
	H5	3.868	3.869	-0.001	3.815	0.053
	H6	3.888	3.885	0.003	3.878	0.010
	H	Chemical shift (ppm)				
		δ (γ-CD)	δ (PM)	Δ δ(CD−PM)	δ (IC)	Δ δ(CD−IC)
	H1	5.124	5.122	0.002	5.115	0.009
	H2	3.684	3.681	0.003	3.670	0.014
C	H3	3.951	3.950	0.001	3.918	0.033
	H4	3.607	3.605	0.002	3.618	−0.011
	H5	3.871	3.869	0.002	3.849	0.022
	H6	3.887	3.885	0.002	3.875	0.012

**Table 2:** Solubility of CRT for each CRT/CD ICs at 25 °C.

Sample	H_2_O (mg/L)	pH6.86 Buffer Salt (mg/L)
CRT/α-CD IC	8402.40 ± 15.72	8032.66 ± 16.23
CRT/HP-β-CD IC	12,429.04 ± 20.33	9125.41 ± 17.92
CRT/γ-CD IC	8607.02 ± 19.08	8270.63 ± 18.52
CRT	1.23 ± 0.07	1.84 ± 0.11

**Table 3:** Pharmacokinetic parameters of CRT and each CRT/CD ICs in SD rats (n = 6). C_max_: peak plasma concentration; T_max_: time to reach highest plasma concentration; T_1/2_: elimination half-life time; AUC: area under the curve; MRT: mean residence time.

	Sample			
	CRT	CRT/α-CD IC	CRT/HP-β-CD IC	CRT/γ-CD IC
C_max_ (μg/mL)	0.545 ± 0.023	2.376 ± 0.118 *	2.487 ± 0.126 *	2.355 ± 0.095 *
T_max_ (h)	2	1	1	1
T_1/2_ (h)	2.059 ± 0.237	2.241 ± 0.131	1.995 ± 0.209	1.928 ± 0.190
AUC_0–12_ (μg·h/mL)	2.411 ± 0.163	8.886 ± 0.115 *	9.522 ± 0.411 *	9.107 ± 0.134 *
AUC_0–∞_ (μg·h/mL)	2.665 ± 0.196	9.723 ± 0.222 *	10.237 ± 0.343 *	9.869 ± 0.245 *
MRT_0–12_ (h)	3.033 ± 0.082	2.787 ± 0.032	2.824 ± 0.034	2.796 ± 0.042
Relative bioavailability (%)		368.561%	394.940%	377.727%

*: *p* < 0.01, significant difference relative to CRT.

## Data Availability

The data presented in this study are available on request from the corresponding author. The data are not publicly available due to innovation.

## Supplementary Materials

The following supporting information can be downloaded at: https://www.mdpi.com/article/10.3390/pharmaceutics15122790/s1. Table S1: Drug encapsulation efficiency of each CRT/CD IC; Figure S1: FT-IR spectra of CRT, CDs, PMs, and ICs at 1800–1400 cm^−1^; Figure S2: DSC curves of CRT, CDs, PMs, and ICs; Figure S3: ^1^H NMR spectra of CRT (DMSO-d_6_), CRT (D_2_O), CDs (D_2_O), PMs (D_2_O), and ICs (D_2_O) at 0–10 ppm; Table S2: Thermodynamic parameters of the three ICs; Figure S4: Molar ratio analyses of ICs using the continuous variation method; Figure S5: Effects of molar ratio time on encapsulation efficiency (n = 3, mean ± S.D.).
