# Supplementary material for: Preparation of trans-Crocetin with High Solubility, Stability, and Oral Bioavailability by Incorporation into Three Types of Cyclodextrins
Source: Pharmaceutics. 2023 Dec 16;15(12):2790. doi: 10.3390/pharmaceutics15122790 (PMC10747661; doi:10.3390/pharmaceutics15122790)
Supplement: Supplementary file 1 [file pharmaceutics-15-02790-s001.zip › pharmaceutics-2695046-supplementary.pdf]

Supporting Information

Preparation of *trans*-Crocetin with High Solubility, Stability, and Oral Bioavailability by Incorporation into Three Types of Cyclodextrins

Nan Liu[1 †], Jie Xiao[2 †], Ling-He Zang[3], Peng Quan[1], Dong-Chun Liu[2*]

[1]School of Pharmacy, Shenyang Pharmaceutical University 110016 Shenyang, Liaoning Province, China

[2]School of Chinese Materia Medica, Shenyang Pharmaceutical University 110016 Shenyang, Liaoning Province, China

[3]School of Life Science and Biopharmaceutics, Shenyang Pharmaceutical University 110016 Shenyang, Liaoning Province, China

*Corresponding author: Dong-Chun Liu (liudc@syphu.edu.cn)

Table S1 Drug encapsulation efficiency of each CRT/CD inclusion complexes.

NO.	EE (%)		
	CRT/α-CD IC	CRT/HP-β-CD IC	CRT/γ-CD IC
1	88.84	89.34	92.37
2	89.80	89.99	91.41
3	88.96	90.47	91.93
Mean	89.20±0.43	89.93±0.57	91.90±0.39
RSD (%)	0.59%	0.63%	0.52%

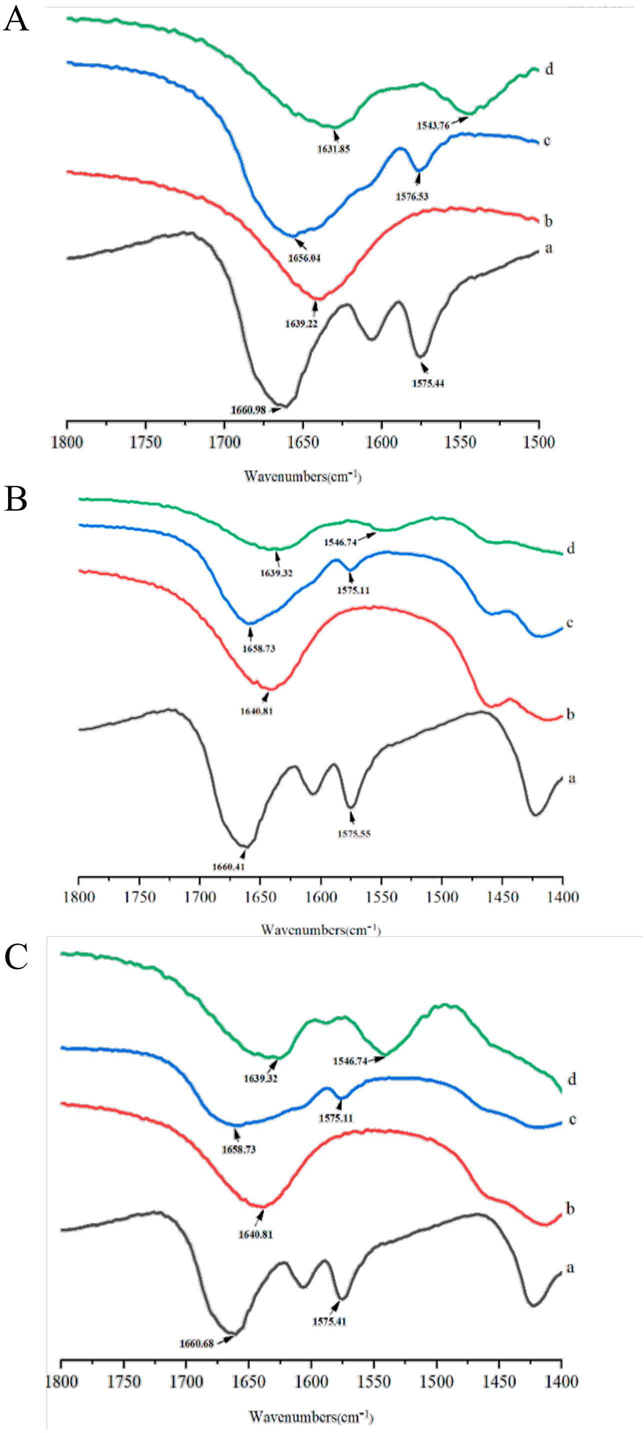

Figure S1. FT-IR spectra of (a) CRT, (b) CD, (c) PM, and (d) IC in CRT/α-CD system (**A**), CRT/HP-β-CD system (**B**), and CRT/γ-CD system (**C**) from 1800-1400 cm^{-1}.

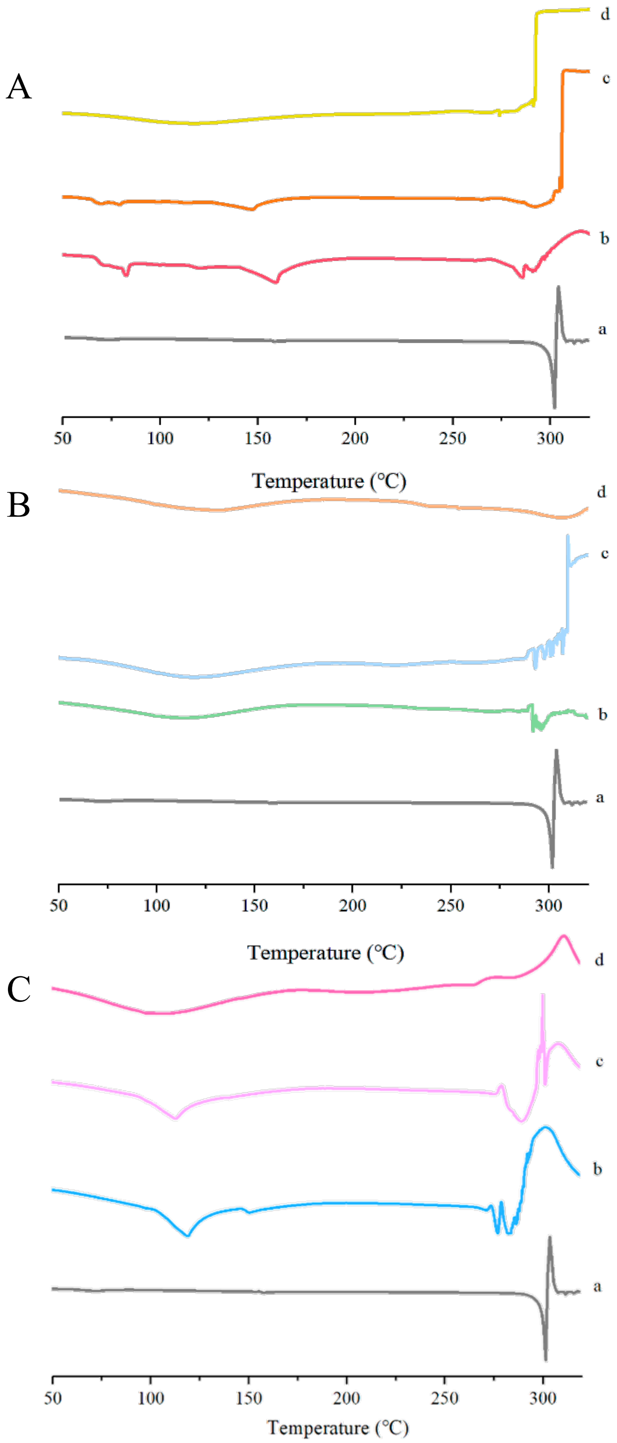

Figure S2. DSC curves of (a) CRT, (b) CD, (c) PM, and (d) IC in CRT/α-CD system (**A**), CRT/HP-β-CD system (**B**), and CRT/γ-CD system (**C**), respectively.

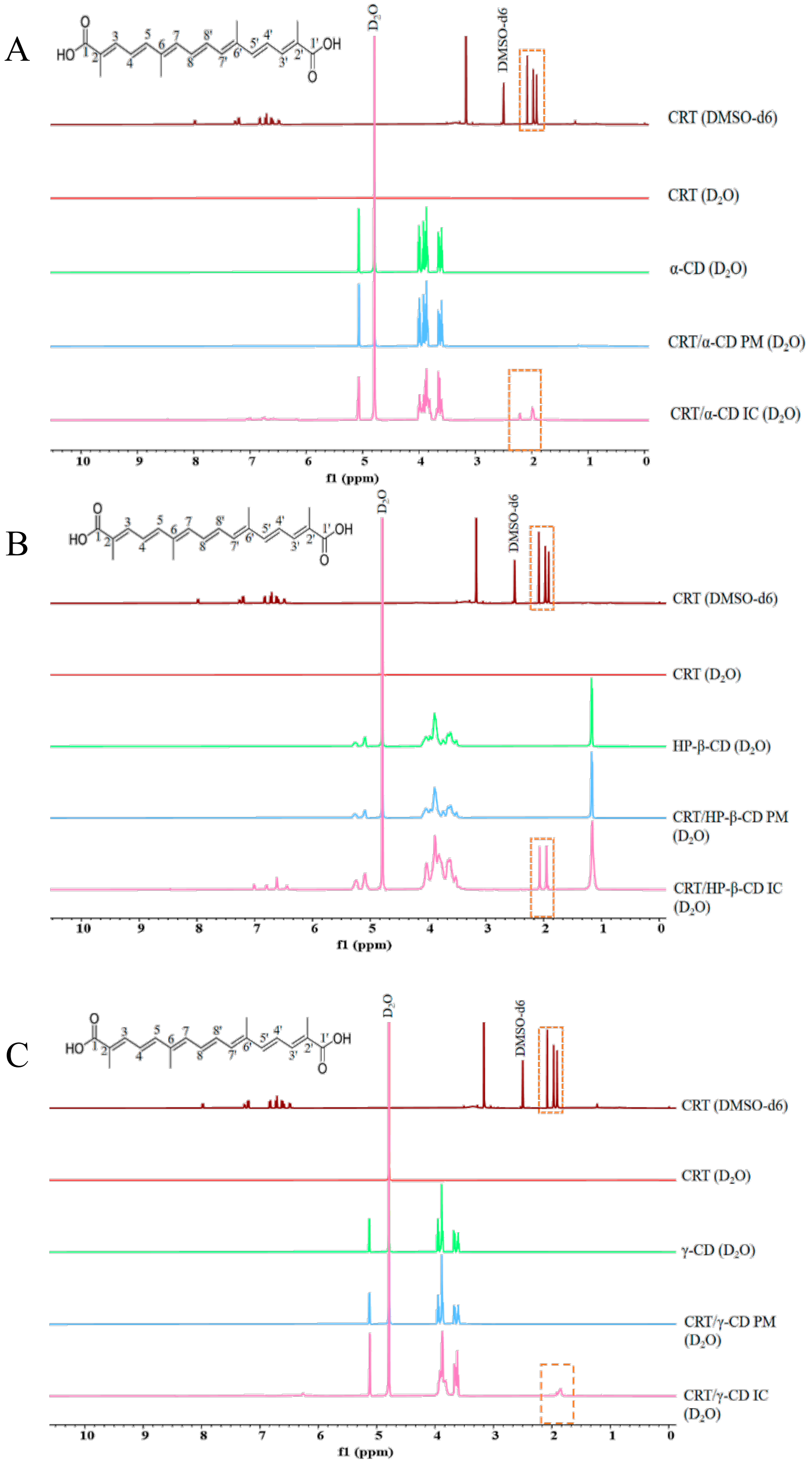

Figure S3. ^1H NMR spectra of CRT (DMSO-d6), CRT (D_2O), CD (D_2O), PM (D_2O), and IC (D_2O) in CRT/α-CD system (**A**), CRT/HP-β-CD system (**B**), and CRT/γ-CD system (**C**) from 0-10 ppm.

Table S2 Thermodynamic parameters of three inclusion complexes.

CD	T (℃)	Equation	△G (KJ)	Ks ($L \cdot mol^{-1}$)
α-CD	37	$Y=0.007x+0.22*10^{-5}$	-20.67	3027.39
HP-β-CD	37	$Y=0.017x-0.22*10^{-5}$	-23.15	7912.04
γ-CD	37	$Y=0.001x+0.22*10^{-5}$	-15.62	427.45

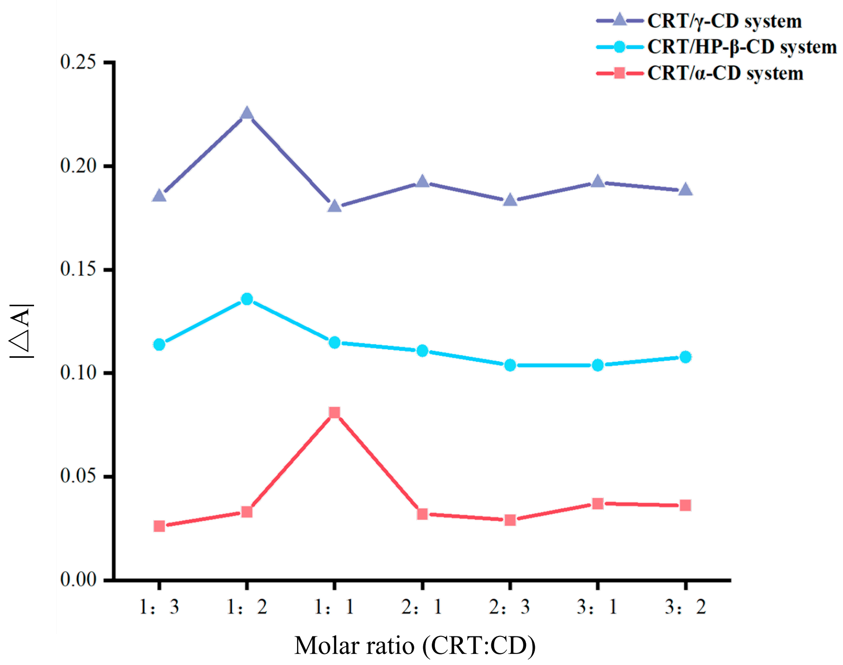

Figure S4. Molar ratio experiments of (■) CRT/α-CD IC, (●) CRT/HP-β-CD IC, and (▲) CRT/γ-CD IC by continuous variation method.

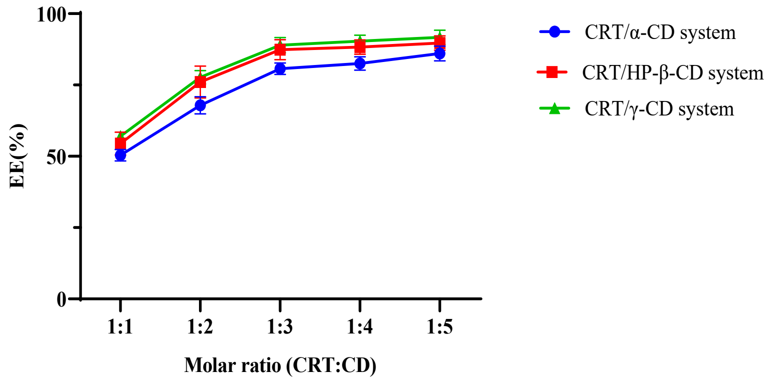

Figure S5. Effects of molar ratio time on encapsulation efficiency (n = 3, mean ± S.D.).
